# Trace Element Distribution in Selected Edible Tissues of Zebu (*Bos indicus*) Cattle Slaughtered at Jimma, SW Ethiopia

**DOI:** 10.1371/journal.pone.0085300

**Published:** 2014-01-21

**Authors:** Veronique Dermauw, Marta Lopéz Alonso, Luc Duchateau, Gijs Du Laing, Tadele Tolosa, Ellen Dierenfeld, Marcus Clauss, Geert Paul Jules Janssens

**Affiliations:** 1 Laboratory of Animal Nutrition, Faculty of Veterinary Medicine, Ghent University, Merelbeke, Belgium; 2 Departemento de Patoloxía Animal, Universidade de Santiago de Compostela, Lugo, Spain; 3 Department of Comparative Physiology and Biometrics, Faculty of Veterinary Medicine, Ghent University, Merelbeke, Belgium; 4 Laboratory of Analytical Chemistry and Applied Ecochemistry Ghent University, Faculty of Bioscience Engineering, Ghent, Belgium; 5 School of Veterinary Medicine, College of Agriculture and Veterinary Medicine of Jimma University, Jimma, Ethiopia; 6 M-team and Mastitis Quality Research Unit, Department of Reproduction, Obstetrics, and Herd Health, Faculty of Veterinary Medicine, Ghent University, Merelbeke, Belgium; 7 Adjunct Faculty, Division of Animal Sciences, University of Missouri-Columbia, Columbia, Missouri, United States of America; 8 Clinic for Zoo Animals, Exotic Pets and Wildlife, Vetsuisse Faculty, University of Zurich, Zurich, Switzerland; Faculty of Animal Sciences and Food Engineering, University of São Paulo, Pirassununga, SP, Brazil, Brazil

## Abstract

The amount of trace elements present in edible bovine tissues is of importance for both animal health and human nutrition. This study presents data on trace element concentrations in semitendinosus and cardiac muscles, livers and kidneys of 60 zebu (*Bos indicus*) bulls, sampled at Jimma, Ethiopia. From 28 of these bulls, blood samples were also obtained. Deficient levels of copper were found in plasma, livers, kidneys and semitendinosus muscles. Suboptimal selenium concentrations were found in plasma and semitendinosus muscles. Semitendinosus muscles contained high iron concentrations. Trace elements were mainly stored in the liver, except for iron and selenium. Cardiac muscles generally contained higher concentrations of trace elements than semitendinous muscles except for zinc. A strong association was found between liver and kidney concentrations of copper, iron, cobalt and molybdenum. Liver storage was well correlated with storage in semitendinosus muscle for selenium and with cardiac muscle for cobalt and selenium. Plasma concentrations of copper, selenium, cobalt were well related with their respective liver concentrations and for cobalt and selenium, also with cardiac muscle concentrations. The data suggest multiple trace element deficiencies in zebu cattle in South-West Ethiopia, with lowered tissue concentrations as a consequence. Based on the comparison of our data with other literature, trace element concentrations in selected edible tissues of *Bos indicus* seem quite similar to those in *Bos taurus*. However, tissue threshold values for deficiency in *Bos taurus* cattle need to be refined and their applicability for *Bos indicus* cattle needs to be evaluated.

## Introduction

Deficiencies in trace elements, such as selenium (Se) and zinc (Zn), are frequently observed in humans in tropical regions such as Ethiopia [1], with severe health consequences (e.g. stunted growth, lowered antioxidant status) [2]. Meat and organ consumption form an important contribution to human nutrition, as these tissues have the capacity to store high amounts of trace elements [3]. However, in Ethiopia, the world's fifth largest cattle holder (FAOSTAT 2013 data, http://faostat3.fao.org), zebu (*Bos indicus*) (*B. indicus*) cattle are typically free ranging on poor pastures and bovine trace element shortages (e.g. copper (Cu) deficiencies) are also very common [4]. Unfortunately, data on trace element concentrations in edible tissues (such as meat, liver, kidney, heart) of *B. indicus* cattle, the most commonly used cattle type in the tropics [5], [6], and more specifically in Ethiopia, are absent.

In *Bos taurus* (*B. taurus*) cattle, the liver is considered the main indicator organ for status evaluation of several essential trace elements, assuming that it forms the main storage depot and is the most responsive tissue to dietary trace element supply [7]. On the contrary, in *B. taurus* cattle, the distribution of trace elements over other tissues, such as muscle is still not well understood. Essential trace elements seem to distribute differently over different types of muscles [8], possibly related to muscle activity and fat content [9]. Furthermore, the relation between liver trace element status and the distribution of trace elements in other tissues is not fully unravelled. A good relationship between liver and muscle cobalt (Co) and Zn concentrations was found in earlier research with cattle with an adequate status [9], whereas such a relationship was not noticed for other trace elements.

For at least some elements (e.g. Cu), especially at lower concentrations, a reasonable link of liver with plasma concentrations is present [10]. Consequently, plasma trace element concentrations are often used as proxy for liver concentrations and thus, trace element status [11]. The relationship between trace element concentrations in plasma and edible tissues, other than liver, however, was not studied before. The latter could be very important for human nutrition, as plasma concentrations might form a more practical tool for early evaluation of trace element concentrations in meat, essential for optimal human health.

Consequently, the objectives of the present study were to: i) present data on trace element concentrations in selected edible tissues and plasma of Ethiopian *B. indicus* cattle and ii) evaluate the association of liver and plasma trace element concentrations, as indicators of trace element status with other tissue concentrations.

## Materials and Methods

### Study area, animals and samples

**Figure 1:**
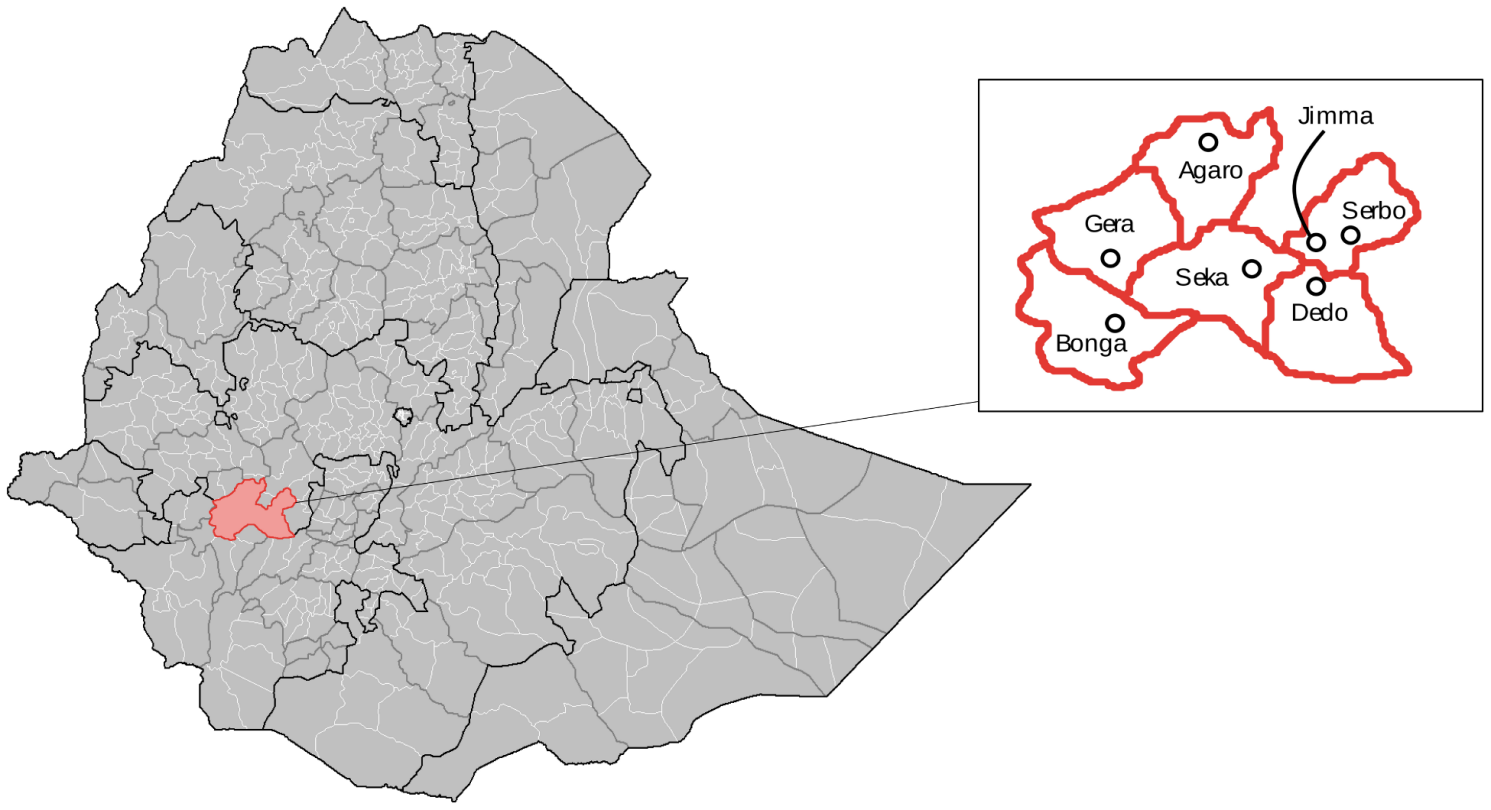
Map presenting the cities of origin of zebu (*Bos indicus*) bulls (*n* = 60) sampled at Jimma, South-West Ethiopia. The red lines depict the woredas, an administrative unit in Ethiopia, to which the cities belong = Bonga (Gimbo), Gera (Gera), Seka (Seka Chekorsa), Agaro (Goma), Jimma, Serbo (Kersa) and Dedo (Dedo).

The study was conducted in Jimma, the largest town in the Gilgel Gibe catchment area, Ethiopia, where bovine trace element deficiencies were previously recognized [4], [12]. The local abattoir receives animals from the urban Jimma zone as well as from surrounding areas and distributes meat and organs within the city of Jimma, for consumption and sale in small restaurants and butcheries. During ten different days, adult zebu (*B. indicus*) bulls (*n* = 60) were randomly selected here, using the lottery method. Their origin was noted and is presented in Figure 1. Thereafter, 28 out of 60 bulls were randomly selected for immediate post-mortem blood sampling, using two sodium heparin tubes (VT-100SH, Venoject®). Subsequently, from all 60 bulls, the cranial part of the left kidney, caudal lobe of the liver, semitendinosus and cardiac muscle (apex of the heart) were sampled. We greatly acknowledge Keraa abattoir and Jimma municipality for their kind permission to sample carcasses. Samples were immediately cooled and transported to the laboratory. Plasma was obtained through centrifugation at 1500× *g* for 10 minutes and excessive fat was removed from tissue samples, where necessary. Samples were stored at −20°C until further analysis.

### Mineral analyses

Muscle, kidney and liver samples were oven dried at 65°C until constant weight and ground through a 2-mm screen. Afterwards, samples were ashed through microwave destruction with 10 ml HNO_3_ (Ultrapure analytical grade for trace element analysis) in open vessels followed by filtration. Finally, all samples were analysed for Zn, Cu, iron (Fe), Se, molybdenum (Mo), Co and manganese (Mn) concentrations through inductively coupled plasma optical emission spectrometry (ICP-OES) (Vista MPX radial, Varian, Palo Alto, USA) and inductively coupled plasma mass spectrometry (ICP-MS) (Elan DRC-e, PerkinElmer, Sunnyvale, CA, USA). All glassware and microwave vessels were pre-rinsed with diluted HNO_3_. A quality control program was employed throughout trace element analyses. Trace element recovery rates from the sampled matrices (plasma, liver, kidney, semitendinosus and cardiac muscle) spiked with two different concentrations of the studied trace elements (in the range of the determined concentrations), were measured. Average recovery was 98%, with a range between 82% (Zn in plasma) and 109% (Mo in kidney). Detection limits in acid digest, determined by the method of Hubaux and Vos [13], were: Mn 0.35 µg/l, Cu 0.25 µg/l, Mo 0.33 µg/l, Se 0.13 µg/l, Fe 21.4 µg/l, Zn 16.4 µg/l and Co 0.14 µg/l. Standards were run frequently alongside samples and all analytical results were blank-corrected.

### Statistical analysis and reference value calculations

To detect whether differences were present for trace element concentrations between liver, kidney, semitendinosus and cardiac muscle, tissue concentrations were compared using a signed rank test at the 5% significance level with Bonferroni's adjustment technique for pairwise comparisons. Median and the first (Q1) and third (Q3) quartiles are reported. Spearman rank correlation coefficients (r) were used to determine the association between liver and plasma and other tissue concentrations of trace elements.

Diagnostic threshold concentrations for *B. taurus* cattle stated in literature [14] and [7] are expressed on wet weight (WW) basis, and in order to compare with the current data, were recalculated to dry weight (DW) basis by multiplying with the conversion factors stated by the authors: 3.5 for liver, 4.5 for other tissues [14]; 3.3 for liver respectively [7].

### Literature search

In order to compare our data with earlier work, we searched for studies mentioning trace element concentrations in the selected tissues in zebu cattle. Based on the zebu distribution maps of Caramelli [5] and Bradley et al. [6], we considered cattle to be zebu-typed if originating from India, Chad, Sudan, Eritrea, Djibouti, Ethiopia, Somalia, Kenya, Tanzania, Mozambique, Malawi or Central African Republic. Other African countries were only partially inhabitated with indicine cattle in addition to crossbreds or even taurine breeds [5], [6], we, therefore, excluded these from our literature search. Furthermore, data from Yemen, Oman and the Emirates were added. Studies performed in Brazil were also included based on the presence of the indicine Nellore and Guzerat breeds [15]. Any other studies specifically stating the use of zebu cattle were also taken into account. Afterwards, studies investigating the same topic in taurine cattle were also searched.

## Results

Summary statistics on trace element concentrations in selected edible tissues and plasma are presented in Table 1 and 2 respectively. Table 3, 4, 5 and 6 demonstrate a comparison of our data with results from literature. Upon comparison of liver concentrations with diagnostic criteria for deficiency in *B. taurus* cattle, 42% of animals (*n* = 60) were considered severely Cu deficient (<19 mg/kg DW; [7]) (Table 1). Plasma Cu concentrations in 29% of animals (*n* = 28) reflected this deficiency (Table 2). Furthermore, semitendinosus muscle and kidney Cu concentrations were below concentrations considered adequate in *B. taurus* cattle in 97% and 100% of animals (*n* = 60). Liver samples did not reveal deficiencies for Fe, Se (*n* = 60; all but one >150, all >0.07 mg/kg DW respectively; [7]) or Co, Zn and Mn (all >0.018, >70 and >3.5 mg/kg DW respectively, [14]). On the contrary, plasma concentrations did indicate a severe Mn and Fe deficiency in 29% and 11% of animals (*n* = 28) respectively. For Se, although none of the animals (*n* = 28) had plasma concentrations below diagnostic thresholds for deficiency according to Suttle [7], 82% of animals had plasma Se concentrations considered at least marginally deficient (<0.06 mg/l), according to Puls [14]. When comparing kidney and muscle concentrations of the latter trace elements with adequate ranges for *B. taurus* cattle, semitendinosus muscle Fe concentrations registered above this range in 73% of animals (*n* = 60), whereas 60% of animals (*n* = 60) had semitendinosus muscle Se concentrations below the adequate range.

**Table 1:** Trace element concentrations (mg/kg DW) in zebu (*Bos indicus*) bull (*n* = 60) tissues sampled at Jimma, Ethiopia with median, first quartile (Q1) and third quartile (Q3) as summary statistics.

	Liver							Kidney							Muscle										
															Semitendinosus				Cardiac						
Element	Median	Q1	-	Q3	Adequate‡			Median	Q1	-	Q3	Adequate‡			Median	Q1	-	Q3	Median	Q1	-	Q3	Adequate‡		
Cu	28.3^a^	7.6	-	65.6	88	-	350	13^b^	12	-	16	18	-	27	2.9^d^	2.0	^-^	3.6	16^c^	15	^-^	17	5.4	-	6.8
Fe	306^a^	245	-	415	158	-	1050	369^a^	286	-	503	135	-	675	80^c^	53	^-^	105	210^b^	194	^-^	239	45	-	54
Mn	13^a^	11	-	14	8.8	-	21	5.2^b^	4.6	-	5.6	5.4	-	9.0	1.1^d^	0.8	^-^	1.8	2.4^c^	1.9	^-^	3.0	2.0	-	3.8
Zn	152^a^	121	-	201	88	-	350	107^b^	94.5	-	126	81	-	113	103^b^	83	^-^	148	81^c^	75	^-^	85		-	
Co	0.47^a^	0.38	-	0.65	0.07	-	0.30	0.39^b^	0.25	-	0.59		-		ND^d^	ND	^-^	ND	0.23^c^	0.14	^-^	0.37		-	
Mo	3.8^a^	3.3	-	4.2	0.49	-	4.9	1.9^b^	1.8	-	2.2	1.0	-	2.6	ND^d^	ND	^-^	0.32	0.33^c^	0.26	^-^	0.42		-	
Se	0.76^b^	0.65	-	0.88	0.88	-	1.8	4.8^a^	4.3	-	5.3	4.5	-	6.8	0.37^d^	0.23	^-^	0.49	0.74^c^	0.59	^-^	0.86	0.32	-	0.68

DW = dry weight.

Adequate range for cattle [14].

^a,b^ Medians sharing a same letter do not differ significantly from each other (p<0.050).

**Table 2:** Trace element concentrations in zebu (*Bos indicus*) bull (*n* = 28) plasma sampled at Jimma, Ethiopia with median, first quartile (Q1) and third quartile (Q3) as summary statistics.

Mineral	Median	Q1	-	Q3	Adequate‡			Threshold value^1^
Cu, mg/l	0.7	0.5	-	0.8	0.8	-	1.5	0.6
Fe, mg/l	1.7	1.2	-	2.0	1.3	-	2.5	1.0
Mn, µg/l	45	18	-	60	6	-	70	20
Zn, mg/l	1.2	1.1	-	1.3	0.8	-	1.4	0.6
Co, µg/l	3.9	2.8	-	5.2		-		0.9^a^
Mo, µg/l	26	19	-	34	10	-	50	100^b^
Se, µg/l	45	36	-	54	80	-	300	20

Adequate range for cattle [14].

^1^ upper threshold value indicating a deficiency risk in *Bos taurus* cattle [7].

^a^ Co: lower boundary of normal Co concentrations in *Bos taurus* cattle [14].

^b^ Mo: lower boundary of Mo concentrations in *Bos taurus* cattle considered elevated [14].

**Table 3:** Literature review of average trace element concentrations in bovine liver (mg/kg WW).

Reference	Country	Cu	Mo	Fe	Zn	Mn	Se	Co
*Bos taurus*								
[20]	Morocco	32	-	-	37	-	-	-
[21] 1	Spain	40	1.1	70	49	2.4	0.2	0.10
[23]	Spain	90	1.4	44	54	3.5	0.2	0.07
[24]	Canada	28	-	-	45	-	0.3	-
[25]	Spain	60	-	-	60	-	-	-
[26]	Jamaica	20	-	-	30	-	0.4	-
[27]	Czech Republic	-	-	-	-	-	0.1	-
[28]	Belgium	80	-	-	40	-	-	-
*Bos indicus* 2								
[29], [30]	Kenya	21	-	-	37	-	0.1	-
[31]	Ethiopia	4	-	293	42	4.1	-	-
[32]	Sudan	67	-	-	-	-	-	-
**This study**	**Ethiopia**	**18**	**1.1**	**118**	**47**	**3.8**	**0.2**	**0.15**

^1^ Geometric mean,

^2^ Presumably *Bos indicus* cattle based upon location or mentioned as such.

**Table 4:** Literature review of average trace element concentrations in bovine kidney (mg/kg WW).

Reference	Country	Cu	Mo	Fe	Zn	Mn	Se	Co
*Bos taurus*								
[20]	Morocco	7.3	-	-	20	-	-	-
[21] 1	Spain	3.1	0.3	51	15	0.7	1.0	0.04
[23]	Spain	4.6	0.5	59	26	1.2	1.4	0.03
[24]	Canada	5.4	-	-	22	-	0.8	-
[25]	Spain	3.7	-	-	22	-	-	-
[26]	Jamaica	3.9	-	-	20	-	1.0	-
[28]	Belgium	5.0	-	-	18	-	-	-
*Bos indicus×Bos taurus*								
[33] 2	Burundi	3.4	-	-	23	-	1.4	-
*Bos indicus*								
**This study**	**Ethiopia**	**3.3**	**0.5**	**97**	**27**	**1.3**	**1.1**	**0.10**

^1^ Geometric mean,

^2^ Presumably *Bos indicus* crossbred cattle based upon location or mentioned as such.

**Table 5:** Literature review of average trace element concentrations in bovine muscle (mg/kg WW).

Source	Country	Cu	Mo	Fe	Zn	Mn	Se	Co
*Bos taurus*								
[8] 1	Spain	0.8	0.13	19	35	0.1	0.10	0.004
[20] 4	Morocco	1.0	-	-	27	-	-	-
[21] b ^,^ 3	Spain	1.7	0.09	39	50	0.2	ND	0.016
[25] 3	Spain	1.3	-	-	53	-	-	-
[27] 3	Czech Republic	-	-	-	-	-	0.04	-
[28] 4	Belgium	1.6	-	-	43	-	-	-
[34] 1	Uruguay	0.4	-	42	25	0.2	0.62	-
[35] a ^,^ 2	Venezuela	0.8	-	19	41	0.3	-	-
[36] a ^,^ 4	USA	0.7	-	20	41	0.1	0.18	-
[37] a ^,^ 2	USA	-	-	17	41	-	-	-
[38] 2	Brazil	-	-	13	34	-	-	-
*Bos indicus×Bos taurus*								
[33] c ^, ^ 4	Burundi	1.1	-	-	54	-	0.20	-
[34] 1	Uruguay	0.6	-	38	24	0.5	0.55	-
[38] 2	Brazil	-	-	13	35	-	-	-
[39] 2	Venezuela	0.9	-	18	38	0.1	-	-
*Bos indicus*								
**This study** 1	**Ethiopia**	**0.7**	**0.07**	**29**	**27**	**0.8**	**0.10**	**0.020**

^a^ Unsure whether *Bos taurus* or crossbred,

^b^ Geometric mean,

^c^ Presumably *Bos indicus×Bos taurus* crossbred cattle based on location,

^1^ Semitendinosus muscle,

^2^ Longissimus dorsi thoracis muscle,

^3^ Diafragm muscle,

^4^ Not-specified.

**Table 6:** Literature review of trace element concentrations in bovine heart (mg/kg WW).

Reference	Country	Cu	Mo	Fe	Zn	Mn	Se	Co
*Bos taurus*								
[8]	Spain	4.4	0.02	45	17	0.3	0.3	0.01
*Bos indicus*×*Bos taurus*								
[33] 1	Burundi	4.0	-	-	20	-	0.3	-
*Bos indicus*								
**This study**	**Ethiopia**	**3.5**	**0.09**	**49**	**18**	**0.6**	**0.2**	**0.06**

^1^ Presumably *Bos indicus×Bos taurus* crossbred cattle based on location.

Liver contained the highest concentrations of trace elements compared to kidney, cardiac and semitendinosus muscle (Table 1), except for Se, of which concentrations were highest in kidney (all p<0.010), and for Fe, for which we found no difference between liver and kidney concentrations (p = 0.035). Cardiac muscles systematically contained higher concentrations of trace elements than semitendinosus muscles, except for Zn of which concentrations were lower in the cardiac muscle samples (all p<0.001, for Mo: p = 0.001). Dry matter concentrations in our study averaged 29% (range: 23–35%) for liver samples, 24% (17–42%) for kidney, 23% (15–31%) for semitendinosus and 22% (18–27%) for cardiac muscle samples.

A strong association was found between liver and kidney concentrations of Cu, Fe, and Co (r = 0.53, r = 0.65, r = 0.80 respectively; all p<0.001) (Table 7), whereas liver and kidney concentrations of Mn, Zn and Mo were weakly correlated (r = 0.28, p = 0.03; r = 0.38, p = 0.003; r = 0.39, p = 0.002 respectively). There was a strong relation between liver and semitendinosus muscle concentrations of Se (r = 0.57, p<0.001) and a weak correlation for Mn (r = 0.36, p = 0.005) whereas for other elements, no significant association were found between these two tissues. Cardiac muscle concentrations of Co and Se were strongly correlated with liver concentrations of the same elements (r = 0.71, r = 0.75, respectively; both p<0.001). Additionally, there was a weak positive association between liver and cardiac concentrations of Fe and Mo (r = 0.28, p = 0.03; r = 0.37, p = 0.003) and a weak negative association for Zn (r = −0.34; p = 0.007).

**Table 7:** Spearman rank correlation coefficient between liver and other tissue concentrations of trace elements in zebu (*Bos indicus*) bulls (*n* = 60) at Jimma, Ethiopia.

		Liver vs.			Plasma vs.		
Element	Plasma vs. liver	Kidney	Muscle		Kidney	Muscle	
			Semitendinosus	Cardiac		Semitendinosus	Cardiac
Cu	0.68***	0.53***	0.01	0.08	0.25	−0.14	0.16
Fe	−0.06	0.65***	0.19	0.28*	0.29	0.11	0.19
Mn	0.42*	0.28*	0.36**	0.19	0.22	0.37	0.16
Zn	−0.39*	0.38**	−0.24	−0.34**	−0.32	0.08	0.18
Co	0.61***	0.80***	0.14	0.71***	0.82***	0.24	0.69***
Mo	0.25	0.39**	0.05	0.37**	−0.05	−0.31	−0.21
Se	0.74***	0.17	0.58**	0.75***	0.15	0.71***	0.83***

p<0.050,

p<0.010,

p<0.001.

Plasma concentrations of Se, Cu, Co were strongly associated with liver concentrations of the same elements (r = 0.74, r = 0.68, r = 0.61; all p<0.001) whereas for Mn and Zn, only a weak relation was present, which was even negative for Zn (r = 0.42, r = −0.39; both p<0.050). For kidney, only Co concentrations were significantly associated with plasma concentrations (r = 0.82, p<0.001). Furthermore, only Se semitendinosus muscle concentrations were associated with plasma concentrations (r = 0.71; p<0.001). Finally, Se and Co cardiac muscle concentrations were strongly related with plasma Se and Co concentrations (r = 0.83, r = 0.69; both p<0.001).

## Discussion

To the best of our knowledge, this is the first study presenting data on trace element concentrations in all selected edible tissues in zebu (*B. indicus*) cattle. Bovine Cu deficiency in this region, as found in previous research [4], [12] was confirmed by both plasma and liver concentrations. On the contrary, it was not clear whether or not a Mn, Fe or Se deficiency was present in the area due to conflicting interpretations based upon plasma and liver concentrations [7], [14], as well as a wide range in threshold values found in literature, mentioned earlier [4]. In this respect, especially threshold values for Se both in liver and plasma vary largely among authors [7], [14], [16]. Thus, our data suggest a multiple trace element deficiency in zebu (*B. indicus*) bulls sampled at Jimma, Ethiopia. Yet, they also point to the clear and urgent need for refinement of bovine plasma and liver thresholds of deficiency, as a practical reference to evaluate the need for supplementation, considering discrepancies were present upon evaluation of plasma and liver concentrations based on diagnostic criteria for deficiency in *B. taurus* cattle [7], [14], [16]. Further, in literature (Table 3, 4, 5 and 6), reported tissue concentrations of Cu concentrations seem to be generally low or at the lower border of adequacy reported by Puls [11], especially in *B. indicus* cattle. On the contrary, in literature, liver, kidney and muscle Fe seem to be at the higher end of or above adequacy ranges stated by the same author [14]. Adequate concentrations are generally observed for Zn, whereas Mn concentrations are rather low and Se often too low in comparison with ranges for adequacy of Puls [14]. This all might point to a generalized trace element imbalance in the cattle sampled in literature. However, it might also indicate that the ranges mentioned by Puls [14] need to be re-evaluated, as they might not reflect bovine trace element status well. The need for clarification is urgent as such reported ranges have a large impact on how we evaluate tissue concentrations in cattle.

An additional hurdle when comparing tissue concentrations with threshold values or other comparative data is the wide variability in dry matter content, both between and within tissues. Because of this, if stated concentrations are expressed in a different unit, e.g. on fresh matter basis, conversion to this unit using a single conversion factor, might create bias. We therefore recommend authors stating data and diagnostic threshold concentrations, to at least mention average dry matter concentrations per tissue.

It also remains unclear whether or not the mentioned thresholds values and adequate ranges are to be extrapolated from *B. taurus* to *B. indicus* cattle as mentioned by Dermauw et al. [12], seeing that even within *B. taurus* cattle, differences in breed sensitivity to deficiency are present [17], [18]. When comparing limited literature data available in *B. taurus* and *B. indicus* cattle, the trace element concentrations in the selected tissues seem similar, although liver and kidney Fe concentrations as well as cardiac Mo concentrations seem higher in *B. indicus* than in *B. taurus* cattle. Moreover, seemingly, tissue Co concentrations are often higher in *B. indicus* than in *B. taurus* cattle (Table 3, 4, 5 and 6). However, to fully unravel potential similarities or differences in trace element distribution between *B. taurus* and *B. indicus* cattle types, further research needs to investigate their trace element storage in a comparable environment during depletion and repletion phases.

Liver contained the highest concentrations of trace elements compared to kidney, cardiac and semitendinosus muscle. Despite significantly higher Se concentrations in the kidney, the liver may still function as a main storage tissue for Se due its larger weight, in agreement with previous research [11]. Fe seemed evenly distributed over liver and kidney, but the tissue weight may again result in the liver being the main storage entity. Liver contained the highest Zn concentrations, but Zn concentrations did not seem to vary widely among liver, kidney and muscle. as observed earlier [19]. Further, the systematically higher trace element concentrations in cardiac than in semitendinosus muscle demonstrate a profound difference in micromineral profile between muscle types. Comparative data in *B. indicus* cattle are absent, and data in *B. taurus* cattle are rare since sampled muscles are often not specified. However, our findings are generally in agreement with earlier research in *B. taurus* cattle [8]. Molybdenum formed an exception, considering the higher concentrations in cardiac muscle as compared to semitendinosus muscle in the current study, which contradicts the earlier data [8]. This may be explained by higher Mo concentrations in the environment and forages in the region [12] leading to a higher accumulation in the *B. indicus* cattle in general and in cardiac muscle more specifically, although none of the sampled tissues contained Mo concentrations beyond the normal ranges stated for *B. taurus* cattle [14]. Overall, the unequal distribution of trace elements amongst the tissues sampled, generally in line with earlier data, relates to the specific function and metabolism of these elements, and i.e. the organ-specific abundance of ligands, such as metallothionein (Cu, Zn uptake) and ceruloplasmin (Cu), binding the trace elements and regulating their uptake [7], [20].

In general, trace element concentrations in the liver, the main storage organ and indicator of trace element status, seemed to correlate reasonably well with storage in other tissues, especially kidney and cardiac muscle. For most trace elements, there was a reasonable association between liver and kidney concentrations, which contradicts previous research [21]. Considering the relation between liver and muscle storage, Blanco Penedo et al. [9] earlier found a strong association for Co and Zn, but not for Se concentrations, the latter being in contrast to our study. Plasma is often presented as a practical sample to assess trace element status [11]. Overall, plasma Cu, Co and Se seemed to associate well with their respective liver concentrations and reasonably well for Mn. This was not found for Fe and Mo or even negative for Zn concentrations. Although based on a small sample size, our data suggest that plasma Co and Se are probably very suitable for evaluation of liver status whereas Cu concentrations were well associated but should probably only be used in the case of expected low liver Cu concentrations [10]. In this regard, the current results discourage the use of plasma Mo, Zn, and especially Fe concentrations, although mentioned previously [11]. Finally, plasma concentrations of Co and Se were well related with cardiac muscle concentrations, which was not reported before.

Overall, our results confirm the differential distribution patterns of Se, Co vs. the other elements, explained by different sites of homeostatic control mechanisms. Homeostatic control of Se and Co is mediated by renal excretion, causing a continuing rise in transport (plasma) and storage (kidney, liver, muscle) pool concentrations, related with rising dietary intake. For the other elements, the intestine is the site of homeostatic control, and consequently, transport and storage pool concentrations might plateau when requirements are met [22].

The lack of information on trace element concentrations in the consumed diets of the sampled *B. indicus* bulls could be considered a limitation of our study. However, in this trial, we focussed on the link between a certain trace element status and the distribution of trace elements in tissues. For liver, it is known that micromineral supply directly affects storage in the organ [7]. Both in literature and in practice, plasma concentrations are often employed as proxy for liver status, whenever sampling of liver is impossible due to practical issues [11]. Indeed, our statements concerning the deficient bovine status in Cu and possibly Se and Mn based on concentrations both in liver and plasma are in line with earlier dietary data from the region, mentioning levels of Cu and Se considered inadequate for *B. taurus* cattle and excessive Fe levels [12]. Considering this multiple trace element deficiency present in the sampled *B. indicus* cattle in the area, we recommend for a future risk analysis study to be performed in order to define both animal and public health risks involved with this condition.

## Conclusion

A multiple trace element deficiency was present in *B. indicus* cattle sampled in South-West Ethiopia. Based on the comparison of our data with literature, zebu cattle seemed to have similar trace element concentrations in edible tissues as *B. taurus* cattle. Overall, the liver was the main storage organ for trace elements and correlated well with concentrations of the same elements in the other tissues. Cardiac muscles generally contained higher amounts of trace elements than semitendinosus muscles. Further, different distribution patterns in edible tissues of Cu, Zn, Mn, Mo and Fe versus Co and Se were observed. Within the ranges observed in our study, plasma values were well related with liver status of Cu, Se and Co and even with muscle Se and Co concentrations.
